# Improving Discharge Rates to Home With the Help of Mobility Technicians: A Step in the Right Direction

**DOI:** 10.7759/cureus.48298

**Published:** 2023-11-05

**Authors:** Swapnil V Patel, Steven Imburgio, Anmol S Johal, Claudia Ramirez, Kristin DiSandro, Divya Mathur, Brian Walch, Vito Buccellato, Mohammad A Hossain, Arif Asif

**Affiliations:** 1 Internal Medicine, Jersey Shore University Medical Center, Neptune City, USA

**Keywords:** quality improvement, cost, discharge, ambulation, mobility technicians

## Abstract

Background

Early ambulation during acute hospitalization has been associated with improved clinical outcomes for patients. Despite the benefits of mobility in the hospital setting, physical therapists and nursing staff are often constrained by time. Mobility technicians (MTs) are individuals with specialized training who have emerged as a potential solution by providing safe ambulation for patients during their hospital stay.

Objectives

The purpose of this quality improvement project was to investigate the impact of MTs on clinical and financial outcomes for admitted patients at a high-volume tertiary institution.

Methods

A quality improvement project was implemented at Jersey Shore University Medical Center, Neptune City, from October 2022 to March 2023. The study was a prospective, single-institution cohort study and included patients admitted to two medical floors. Patients were divided into an experimental group that received services from MTs and a control group that did not receive this service but was eligible based on clinical status. The primary endpoint was the proportion of patients discharged to home. Secondary outcomes included the length of stay and financial impact.

Results

A total of 396 admitted patients were included, with 222 patients in the MT group and 174 in the non-MT group. Patients in the MT group were discharged home more frequently, at a rate of 79.7% compared to 66.1% for patients in the non-MT group (p = 0.002). MTs contributed to an average 2.4-day reduction in the length of hospital stay (7.8 days vs. 10.2 days, p = 0.007). The MT intervention led to an estimated net savings of $148,500 during the six-month study period. Additionally, 2.9 daily hospital beds were created.

Conclusion

Implementing an MT program significantly increased the discharge-to-home rates and decreased hospital length of stay. Preliminary analysis suggests that this intervention is cost-effective and can assist institutions in managing increased hospital capacity strain through the creation of additional hospital beds.

## Introduction

Over the past few decades, the global population has aged rapidly [1], which has placed a considerable burden on healthcare systems [2]. A significant proportion of this demographic of elderly hospitalized patients is frail, predisposing those individuals to worse clinical outcomes [3] due to the inherent vulnerability associated with frailty. Scoring systems for frailty have served as useful prognostic indicators to predict discharge destinations, with frail patients experiencing higher rates of discharge to skilled nursing facilities [4]. While some patients may benefit from rehabilitation placement, there are also potential risks, including delays in care, increased falls, medication or treatment errors, and equipment failures [5]. Despite increased reliance on post-acute care facilities, recent studies have shown that patients who are discharged home with home health services have decreased rates of 30-day readmissions, reduced short-term mortality, and an improvement in activities of daily living [6]. In light of these data, there has been a national initiative to safely discharge a higher percentage of patients home [7]. Institutions are now focusing on early ambulation as a way to optimize the functional status of patients, since prolonged immobilization has been associated with higher utilization of skilled nursing facilities [8]. With older patients being subject to a high degree of deconditioning from extended periods of bed rest [9], there are often substantial challenges in ensuring patient mobility in the inpatient setting by both physical therapists and nurses. These factors include staffing issues, time constraints, growing patient loads, miscommunication regarding roles, and associated costs [10-12].

Mobility technicians (MTs) are individuals with specialized training in the ambulation of patients. The implementation of MTs in the inpatient setting has emerged as a potential novel solution to decrease the number of patients discharged to rehabilitation facilities. For example, Novack et al. demonstrated that MTs increased the rate of discharge to home for postoperative total knee arthroplasty patients from 81.5% to 90.5% [13]. While MTs have become increasingly implemented, there is a scarcity of literature regarding the effectiveness and potential cost savings of this intervention. As a result, we introduced MTs at our high-volume tertiary institution and prospectively analyzed various clinical outcomes including rates of discharge home, in addition to the financial implications of piloting an MT program. The overall aim of this quality improvement study was to investigate the clinical and economic impact of implementing MTs in the inpatient setting.

## Materials and methods

Quality improvement process

To combat increasing hospital strain, a multidisciplinary quality improvement team at Jersey Shore University Medical Center (JSUMC) was created. The team consisted of hospital administration, physician leaders, nursing leaders, physical therapists, patient care associates, and the quality improvement department. Previously, this committee identified that a large percentage of hospitalized patients were pending placement to rehabilitation facilities, contributing to both decreased patient mobility and bed turnover rates. A quality improvement proposal was subsequently created to increase patient mobilization with the use of MTs in an effort to reduce discharge rates to home.

Setting

JSUMC is a 630-bed tertiary teaching medical center in central New Jersey with seven residency and seven fellowship programs. Excluding psychiatry, women’s health, and pediatric services, there are 464 beds. The participants in this study were located in two telemetry units, which did not include psychiatric, women’s health, or pediatric patients.

Overview of the project

Adults 18 years and older admitted to our medical center or under observation status were included in the study. Specifically, the quality improvement project was conducted as a randomized two-arm study. Data were collected in a prospective fashion. This quality improvement study was determined to be exempt from Institutional Review Board oversight because patient-level data were not collected.

Interventions

Two telemetry-based units with comparable patient profiles were selected to initiate the program. These specific floors were chosen following a thorough analysis of internal data from the entire year of 2022, which had previously indicated similar patterns in age, inpatient mortality, 30-day readmission rates, and length of stay for admitted patients (Table 1). 

**Table 1:** Comparison of Patient Profiles From the Two Telemetry Floors

Clinical Metric	Telemetry Floor #1	Telemetry Floor #2
Average age	65.8 years	65.2 years
Inpatient mortality	0.42%	0.39%
30-day readmissions	0.15%	0.13%
Average length of stay	6.0 days	6.1 days

MTs were hired for these units after completing a standardized training course. They each received 12 hours of training in total over the course of one week. The training was conducted under the guidance of licensed physical therapists and registered nurses. The training course included assessment of abnormal vital signs, lifting and mobility techniques, and safe transfer skills. Training also encompassed the safe and proper use of assistive equipment with guidance on educating the interprofessional team and family on the importance of early mobility. These MTs were implemented over a six-month period from October 2022 through March 2023.

Nursing staff were responsible for identifying previously mobile patients who were at high risk for hospital deconditioning and who might benefit from an out-of-bed and ambulation program. Patients who were interested and met the study inclusion criteria provided written informed consent and were subsequently randomized into one of the two study arms. The control group received standard mobilization with nursing and physical therapy staff, while the experimental group received these services and was also scheduled to work with MTs. Patients in the MT group were offered one mobility session every day, which lasted up to 20 minutes in duration. 

Inclusion criteria

The inclusion criteria for patients include the following: (1) adult patients ≥ 18 years of age, (2) those admitted to one of the study telemetry floors, (3) an activity level of “Up as tolerated” or “Up with assistance” as ordered by the primary physician, (4) assessment if a physical therapy consult is needed prior to MT intervening as determined by the primary physician, and (5) patients requiring no more than minimal assistance required for ambulation at baseline as screened by the patient's primary nurse.

Exclusion criteria

The exclusion criteria included patients downgraded from the medical, cardiovascular, surgical, or neurological intensive care units.

Primary outcome

The primary outcome of this study was the rates of discharge to home. 

Secondary outcomes

Secondary endpoints included the length of stay and the financial impact. 

Statistics

Analysis of the data involved descriptive statistics, the Mann-Whitney U test, the chi-square test, and the Fisher exact test. Data was summarized as median (interquartile range [IQR]) and percentages. Statistical analyses were performed using IBM SPSS Statistics for Windows, Version 24 (Released 2016; IBM Corp., Armonk, New York, United States). An alpha (p) value ≤ 0.05 was considered statistically significant.

## Results

Rates of discharge to home

During the study period, 396 individuals were admitted to the two medical units with home being the disposition for 292 of these patients (73.7%). A total of 222 patients participated in the MT group and 174 patients did not utilize MT assistance. Patients in the MT group were more frequently discharged home at a rate of 79.7% (n = 177) as compared to 66.1% (n = 115) in the non-MT group (p = 0.002).

Length of stay

The median length of stay for all patients in the study was 8.3 days. Patients who worked with the mobility helpers had a reduced median length of stay of 7.8 days as compared to the median length of stay of 10.2 days for patients who did not use these services (p = 0.007).

Financial impact

Patients who worked with mobility helpers experienced a reduced length of stay by 2.4 days. With a conservative estimate of hospital stay cost at our institution at $300 (U.S. dollars) per day [14], there was an estimated savings of around $159,840 just during the study period for the 222 patients in the mobility helper group. On average, each patient who worked with the MTs saved $720. The mobility helpers collectively worked 756 hours at an average salary of $15 per hour for a total cost of $11,340 during the study time period. Overall, the MTs would need to successfully treat only 15.8 patients over the 6 months to recover savings equivalent to their salary.

The overall projected net savings of the mobility helpers for the 6-month time period of our study was $148,500, which translates into an estimated annual savings of $297,000 if the study was continued for a full year at the same rate. Furthermore, for the 222 patients in the intervention group with a reduced length of stay of 2.4 days over the 6 months and an occupancy rate of 100%, an estimated 2.9 daily hospital beds were created. Assuming that these findings are consistently seen over the course of a full year, 1,054.9 projected hospital beds could have been created for patients.

**Table 2:** Comparison of Results From the MT and Non-MT Groups MT: mobility technician

	MT (n = 222)	Non-MT (n = 174)	Total (n = 396)
Discharge rates to home	79.7% (n = 177)	66.1% (n = 115)	73.7% (n = 292)
Average length of stay	7.8 days	10.2 days	8.3 days

## Discussion

The most important finding of this study is that patients who ambulated with MT assistance experienced increased rates of discharge to home. While prior literature has demonstrated similar findings, the interpretation and generalizability of these results are limited due to the highly specific patient demographics, which included primarily orthopedic surgery patients [13, 15], COVID-positive patients [16], or intensive care unit patients [17]. This study is one of the few to demonstrate that an early mobilization protocol led by MTs can reproduce these results with a diverse, heterogeneous patient population. Only one other study that implemented dedicated MTs demonstrated no significant increase in discharge rates to home between the MT and non-MT groups. Differences in study design may explain these contrasting findings, with Hamilton et al. using a smaller patient population of just 50 patients in the intervention group, of which 26% never received any daily ambulation [18].

Despite the increased adoption, there is a scarcity of data regarding the impact of MTs on the length of stay; this study demonstrates a reduction in this metric. To date, only two studies have analyzed the impact of MTs on median length of stay, with both showing no significant change [13, 15]. However, an important consideration for these contrasting results is their inclusion criteria, which selected for elective orthopedic surgery patients with expected short hospital courses. This study is the first to report a reduced length of stay in a diverse population of hospitalized adult patients following the introduction of MTs. The clinical importance of reducing the length of stay is that it allows hospitals to improve bed turnover rates and subsequently reduce capacity strain [19]. Another benefit is the decreased frequency of inpatient complications since an increased length of stay is associated with higher rates of infections, falls, and venous thromboembolism [20]. This suggests that MT-led ambulation may be a safe alternative to traditional physical therapy mobility programs.

Immobility in hospitalized adults is highly prevalent and associated with increased adverse outcomes, particularly in older patients. Reduced mobility levels in the inpatient setting have been linked to increased mortality, frequent readmissions, longer lengths of stay, and higher rates of discharge to skilled nursing facilities [9, 21, 22]. In response to the overwhelming data highlighting the benefits of early ambulation, many institutions have piloted programs in recent years to introduce staff who can mobilize patients during their hospitalization [13, 15-18, 23-25]. Although it is challenging to discharge all patients to home, the use of MTs can help maintain and improve patients' functional status for their potential return to home.

In addition to improved clinical outcomes for hospitalized patients, the implementation of an MT program in the inpatient setting can produce substantial savings. Preliminary cost analysis has shown that each patient in the MT group accounted for an average saving of $720, derived solely from the reduced hospital length of stay. Overall healthcare costs can be further reduced through this intervention due to lower utilization of post-acute care services, decreased rates of hospital-associated complications, the creation of additional hospital bed availability, and decreased reliance on inpatient physical therapists. Mazzei et al. estimated that MTs would need to successfully treat just five patients annually to offset the costs equivalent to their salary [15]. Although MTs are a cost-effective resource for institutions aiming to help patients achieve their mobility goals, further research is required to fully understand the costs associated with launching a successful hospital-wide MT-led ambulation program.

Given the deconditioning associated with acute hospitalizations, optimizing the functional status of patients during their hospital stay is essential. MTs can effectively improve the physical condition of hospitalized patients through safe ambulation. The addition of this role to an existing interprofessional medical team can have a positive impact on clinical metrics by decreasing the number of patients discharged to rehabilitation facilities and by reducing the hospital length of stay. Beyond their clinical value, MTs demonstrate economic benefits through significant cost savings for healthcare institutions. Further prospective studies are necessary to establish specific guidelines for the successful implementation of a mobility program and to determine how to effectively scale MTs across an entire hospital system.

Limitations

There are several limitations to our study. First, the single-center study design may limit the generalizability of our results to other patient populations at different institutions. Additionally, this pilot study was conducted on two telemetry floors, resulting in a small sample size and not addressing the feasibility of expanding the MT program across an entire hospital system. Another significant limitation is the reliance on nursing staff to screen patients for potential benefits from MT assistance, which may have led to a non-random selection of participants. Lastly, potential complications were not analyzed; this is concerning as increased ambulation may increase the risk of falls.

## Conclusions

The addition of the Mobility Technician (MT) role in the inpatient setting is an effective way to promote early ambulation among hospitalized adult patients. An MT-led mobility program has the potential to increase the proportion of patients discharged home and reduce hospital length of stay. This, in turn, can create additional hospital beds and reduce reliance on traditional services for patient mobility, potentially resulting in significant cost savings. In summary, the implementation of a mobility program can improve clinical outcomes for admitted patients and be cost-effective for healthcare institutions.
